# The role of murine leukemia virus glycosated Gag protein in virus replication

**DOI:** 10.1186/1742-4690-8-S2-O20

**Published:** 2011-10-03

**Authors:** Hung Fan

**Affiliations:** 1 Department of Molecular Biology and Biochemistry, and Cancer Research Institute, University of California, Irvine, CA 92697-3905, USA

## 

Murine leukemia viruses (MuLVs) encode an alternate form of Gag protein in addition to the standard Gag precursor polyprotein, glycosylated Gag (glyco-gag or gPr80*^gag^*). gPr80*^gag^* contains additional amino-terminal peptides, and it is glycosylated. Until recently the function of gPr80*^gag^* was unclear, but we have recently shown that gPr80*^gag^* facilitates viral budding and release through lipid rafts. Glyco-gag mutant virus is less efficiently released from fibroblasts, mutant virus has lower cholesterol content (and higher buoyant density), and less Gag precursor polyprotein (Pr65*^gag^*) is associated with detergent-resistant membranes in infected cells. The mechanism by which gPr80*^gag^* facilitates virus release is under investigation. The N-terminal 88 amino acids of gPr80*^gag^* are sufficient to enhance virus release, indicating that the Gag sequences on this protein are not required. Cellular La protein is involved in gPr80*^gag^* function, since over-expression of La phenocopies gPr80*^gag^* in facilitating virus release, and knockdown of La abrogates gPr80*^gag^*-enhancement of virus release. MuLV glyco-gag can also facilitate release of HIV-1 particles from transfected cells, which suggests that these two viruses may share mechanisms for directing virus release through lipid rafts. gPr80*^gag^*also counteracts the murine APOBEC3 restriction factor (mA3) since gPr80*^gag^*mutant virus shows a defect for establishment of infection in wild-type mice, but not in mA3 knockout animals.

